# Mediterranean Diet Adherence in People With Parkinson's Disease Reduces Constipation Symptoms and Changes Fecal Microbiota After a 5-Week Single-Arm Pilot Study

**DOI:** 10.3389/fneur.2021.794640

**Published:** 2021-12-23

**Authors:** Carley Rusch, Matthew Beke, Lily Tucciarone, Carmelo Nieves, Maria Ukhanova, Massimiliano S. Tagliamonte, Volker Mai, Joon Hyuk Suh, Yu Wang, Shannon Chiu, Bhavana Patel, Adolfo Ramirez-Zamora, Bobbi Langkamp-Henken

**Affiliations:** ^1^Food Science and Human Nutrition Department, University of Florida, Gainesville, FL, United States; ^2^Department of Neurology, Norman Fixel Institute for Neurological Diseases, University of Florida, Gainesville, FL, United States; ^3^Department of Epidemiology, Emerging Pathogens Institute, University of Florida, Gainesville, FL, United States; ^4^Department of Pathology, Immunology and Laboratory Medicine, Emerging Pathogens Institute, University of Florida, Gainesville, FL, United States; ^5^Food Science and Human Nutrition Department, Citrus Research and Education Center, University of Florida, Lake Alfred, FL, United States

**Keywords:** Parkinson's disease, Mediterranean diet, constipation, microbiota, intestinal permeability

## Abstract

**Introduction:** Non-motor symptoms of Parkinson's disease (PD) such as gastrointestinal (GI) dysfunction are common, yet little is known about how modifying dietary intake impacts PD symptoms. The aim of this study in individuals with PD was to determine whether a Mediterranean diet intervention is feasible and affects GI function, intestinal permeability and fecal microbial communities.

**Methods:** A single-arm, 5-week Mediterranean diet intervention study was conducted in eight people with PD. Daily and weekly questionnaires were administered to determine changes in GI symptoms. Urine and stool samples were collected at baseline and after 5 weeks to assess intestinal permeability and fecal microbial communities. Additionally, live-in partners of the participants with PD were matched as controls (*n* = 8) for baseline urine and stool samples.

**Results:** Participants with PD increased intake of Mediterranean diet based on adherence scores from baseline to week 5 (4.4 ± 0.6 vs. 11.9 ± 0.7; *P* < 0.01 with >10 representing good adherence), which was linked with weight loss (77.4 kg vs. 74.9 kg, *P* = 0.01). Constipation syndrome scores decreased after 5 weeks (2.3 ± 0.5 vs. 1.5 ± 0.3; *P* = 0.04). *Bilophila*, was higher at baseline in PD (0.6 ± 0.1% vs. 0.2 ± 0.1% *P* = 0.02) and slightly decreased after the diet intervention (0.5 ± 0.1%; *P* = 0.01). Interestingly, the proportion of *Roseburia* was significantly lower in PD compared to controls (0.6 ± 0.2% vs. 1.6 ± 0.3%; *P* = 0.02) and increased at week 5 (0.9 ± 0.2%; *P* < 0.01). No differences were observed for markers of intestinal permeability between the control and PD groups or post-intervention.

**Conclusions:** Short-term Mediterranean diet adherence is feasible in participants with PD; correlated with weight loss, improved constipation, and modified gut microbiota.

**Clinical Trial Registration:** ClinicalTrials.gov, identifier: NCT03851861.

## Introduction

Parkinson's disease (PD), is characterized by the accumulation of alpha-synuclein and loss of dopaminergic neurons resulting in motor and non-motor manifestations (1). Non-motor symptoms of PD often occur several years before onset of other symptoms including olfactory, cardiovascular and gastrointestinal (GI) dysfunction (2). Constipation, a common GI symptom prevalent in up to 80% of individuals with PD, often leads to reduced quality of life and increased disease burden (3–5). GI dysfunctions, including maintenance of the intestinal barrier and microbial diversity, have been an emerging area of interest in neurodegenerative research, yet little is known about how modifying dietary intake may impact these outcomes in individuals with PD.

The intestinal barrier is a dynamic system containing of epithelial cells, mucus secreting goblets cells and cytokine secreting immune cells. Digestive enzymes, as well as microbial metabolites, contribute to absorption of necessary nutrients, electrolytes, and water while barrier fortification ensures that potentially harmful pathogens and toxins remain within the luminal compartment (6). Both, intestinal permeability as well as local and systemic inflammation have been shown to be altered in PD. Two studies yielding conflicting results have investigated intestinal permeability in PD patients compared to matched controls using an orally administered sugar probe (7, 8). Other studies have found alterations in tight junction proteins (e.g., occludin and zonulin) and increased intestinal inflammation (e.g., fecal calprotectin, lactoferrin) which can lead to increased intestinal permeability (9–11). The results of these pilot studies warrant further investigation to not only confirm their findings, but also establish if diet interventions can promote beneficial microbes and improve barrier function in people with PD.

The Mediterranean diet includes (1) variety of fruits, vegetables, legumes, unrefined cereals, (2) healthy dietary fats from olive oil, avocadoes, nuts and seeds, (3) moderate amounts of fish, poultry, eggs and dairy, and (4) limited intake from red meats or sweets (12). Fiber is an under-consumed component of the Western diet enriched in the Mediterranean diet that helps maintain regular stool frequency, thereby reducing symptoms of constipation. Prebiotic fibers found naturally in fruits and vegetables and added to many foods are selectively fermented by intestinal bacteria to produce short chain fatty acids (e.g., acetate, butyrate, propionate) important for maintaining intestinal barrier function and reducing intestinal inflammation (13, 14). Higher adherence to the Mediterranean diet has been associated with increased: (i) microbial richness (15), (ii) microbes thought to be beneficial (15–17), and (iii) increased short chain fatty acids (15, 17, 18). Recent studies have shown distinct alterations in intestinal bacteria and bacterial products with PD, such as decreased *Faecalibacterium* (butyrate producer), *Prevotellaceae*, and overall reduced short chain fatty acids compared to healthy controls (19, 20). To date, there have been limited therapeutic diet interventions aimed to specifically improve GI dysfunction in PD patients.

Therefore, the purpose of this study was to determine whether adhering to a Mediterranean diet is feasible and induces beneficial changes in GI function (e.g., stool frequency and consistency and GI symptoms), intestinal permeability and fecal microbial communities in individuals with PD.

## Materials and Methods

### Participants

Study participants were recruited at the University of Florida Norman Fixel Institute for Neurological Diseases in Gainesville, FL. This study was approved by the University of Florida Institutional Review Board-01 and was carried out in accordance with the guidelines of the Declaration of Helsinki. To compare differences in intestinal permeability and microbiota at baseline and after the diet intervention, live-in partners of the participants with PD were recruited to provide urine and stool samples during the baseline period (control group). Participant inclusion and exclusion criteria are provided in the Methods and Results in Supplementary Material.

### Study Design and Questionnaires

Participants with PD were enrolled in a 2-week baseline period followed by a 5-week diet intervention period from April to September 2019. Participants were instructed to maintain their usual diet and level of physical activity throughout the baseline period and completed questionnaires. Daily questionnaires assessed stool frequency (i.e., number of weekly bowel movements), stool form using the Bristol Stool Scale (21) and adverse events. The weekly questionnaire assessed GI symptoms using the Gastrointestinal Symptom Rating Scale (GSRS) (22). During the baseline period, participants with PD completed 3, 24-h dietary recalls (2 weekday and 1 weekend) using the Automated Self-Administered 24-h Dietary Assessment Tool, version 2018 (23). On the last day of the baseline period, participants with PD and their live-in partners attended their first study visit (*visit 1*). Study staff measured height (seca 213, Hamburg, Germany), weight (seca 813, Hamburg, Germany) and Mediterranean diet adherence using the 14-item Mediterranean diet adherence screener (MEDAS) where a score ≥10 indicates “good adherence” (24). Measurements completed at visit 1 were repeated at the end of the 5-week intervention period for the participants with PD (*visit* 2).

### Neurological Evaluation

At visit 1, a movement disorders trained neurologist determined baseline motor and non-motor symptoms using the Movement Disorder Society-Unified Parkinson's Diseases Rating Scale (MDS-UPDRS) (25) and cognitive function using the Montreal Cognitive Assessment (MoCA) (26). Participants were asked to take their usual medications and evaluated during the “ON” period of their medication, when possible. PD medications did not change during the study protocol. Participants in the control group were evaluated by the neurologist to rule out signs of neurological disease or condition.

### Mediterranean Diet Intervention

At the first study visit, study dietitians provided diet education and instructed participants with PD to increase their adherence to a Mediterranean diet for 5-weeks. Participants were followed weekly by study dietitians (4 times) via telephone to support adherence during the intervention (i.e., diet compliance, areas of improvement, goal setting and participant questions). Guidelines for the Mediterranean diet were developed based on the Prevención con Dieta Mediterránea (PREDIMED) trial protocol (27) and participants used the MEDAS as a food diary to improve their diet adherence. In brief, the diet includes: (a) abundant use of olive oil; (b) consumption of ≥2 daily servings of vegetables; (c) ≥2–3 daily serving of fresh fruits (including 100% juice); (d) ≥3 weekly servings of legumes; (e) ≥3 weekly servings of fish or seafood (at least one serving of fatty fish); (f) ≥3 weekly servings of nuts or seeds; (g) select white instead of red meats or processed meats; and h) cook at least twice a week with a sofrito sauce. *Ad libitum* consumption was allowed for the following food items: nuts, eggs, fish, seafood, low-fat cheese and whole-grain cereals. For usual drinkers, the main source of alcohol should be wine. Participants were instructed to limit consumption of cream, butter/margarine, red and processed meats, carbonated and sugared beverages, commercial bakery products and desserts, French fries or potato chips.

### Sample Collection

#### Stool Samples

Stool samples were collected during the second week of the baseline period (PD and control groups) and at week 5 of the intervention period (PD group only) using a commode specimen collection system (Biomedical Polymers, Inc.) for fecal microbiota analyses. Participants were instructed to portion ~1 g of stool sample into tubes containing 3 mm glass beads and 7 ml RNA*later* Stabilization Solution (Invitrogen) to preserve the sample at room temperature. Samples were shipped overnight by participants to the study site and stored at −80°C until processing.

#### Urine Samples and Permeability Testing

Urine samples were collected at visit 1 (PD and control groups) and visit 2 (PD group only) as described by van Wijck et al. using orally administered sugars as markers of intestinal permeability (28). In brief, after an 8-h fast, participants arrived at the study site, emptied their bladder, and drank a beverage containing five sugars. After drinking the study beverage, participants provided a fasted 5-h urine collection at the study site, followed by an unfasted 19-h collection at home to complete a full 24-h urine collection. Urine samples were kept on ice packs in coolers. Participants returned the 19-h urine collection to the study site the following day. Urine collections from each timepoint (0–5 h and 5–24 h) were weighed and stored at −80°C until analysis.

The 5-sugar beverage contained 1.0 g sucrose (Now Real Food, IL, USA), 0.5 g L-rhamnose (Yundao Production, China), 1.0 g lactulose (Wockhardt, IL, USA), 1.0 g sucralose (Micro Ingredients, CA, USA), and 1.0 g erythritol (Now Real Food, IL, USA) in 150 ml water. However, when analyzing urinary concentrations of the sugars at the completion of the study it was determined that L-rhamnose was mispackaged and actually contained neotame (Methods and Results in Supplementary Material). Neotame is a non-nutritive, U.S. Food and Drug Administration-approved food additive (29). Subsequently, erythritol was used for the ratio for upper GI permeability because it is of similar molecular weight to L-rhamnose (122.1 vs. 164.2 g/mol, respectively). Urinary recovery and permeability ratios were calculated as previously described (30). Urinary output of sucrose (0–5-h urine collection) assessed gastroduodenal permeability, the ratio of the concentration of lactulose/erythritol in the 5-h urine collection assessed upper GI permeability, the ratio of sucralose/erythritol (5–24-h collection) assessed colonic permeability, and the ratio of sucralose/erythritol (0–24-h collection) assessed whole gut permeability.

### Laboratory Assays

#### Intestinal Permeability LC-MS/MS Analysis

Liquid chromatography with tandem mass spectrometry (LC–MS/MS) analyses were carried out on urine samples using an Ultimate 3000 LC system coupled to a TSQ Quantiva triple quadrupole mass spectrometer (Thermo Fisher Scientific, San Jose, CA, USA) with hydrophilic interaction chromatography. Sample preparation and LC-MS/MS procedure are described further in Methods and Results in Supplementary Material.

#### DNA Isolation and Sequencing for 16S rDNA and qPCR Analyses

Bacterial genomic DNA for 16S sequencing was isolated from 1 g stool sample. The QIAamp DNA Stool Mini kit was used according to the manufacturer's instructions with an added 3-min bead beating step after the addition of the first lysis buffer (31). Methods for 16S RNA gene sequencing were conducted as previously described (32). DNA products were purified using Mag-Bind TotalPure NGS beads (Omega BioTek) according to the manufacturer's protocol and were quantified using a Quanti-iT dsDNA assay kit (Invitrogen). Equimolar amounts of DNA were pooled and submitted to an on-campus core facility for sequencing using the Illumina MiSeq platform (250 bp paired-end reads). Sequences with a length of <200 nucleotides and single stranded reads were removed from the analysis. Quantitative Insights into Microbial Ecology 2 (QIIME2) tool (version 2019.7; open source software) (33) pipeline was used to trim and quality filter sequences. Amplicon Sequence Variants (ASVs) were determined using Deblur (34) and clustered into *de novo* operational taxonomic units (OTUs) at the 95% and 98% similarity level using VSEARCH (35). Rarefaction curves to assess species richness and comparability of the samples were also generated through these pipelines. Chimeras were removed, and taxonomy was assigned to OTUs using a classifier built with Scikitlearn (36) and verified against the Greengenes 16S rRNA gene database (Version 13.8) (37). A phylogenetic tree for diversity analyses was generated with the FastTree pipeline (38). Quantative polymerase chain reaction procedure is further described in Methods and Results in Supplementary Material.

### Statistical Methods

#### Questionnaire and Intestinal Permeability Analyses

Participants with PD were matched with their healthy partner and compared for differences using a paired *t*-test for demographics, urine and stool sample analyses. Questionnaires and sample analyses for the intervention arm (PD group only) were compared using paired *t*-test between baseline and week 5 measurements. If normality failed, the non-parametric equivalent was used (i.e., Wilcoxon Signed Rank test). Statistical analyses were carried out using Sigma Plot Version 14.0 (Systat Software Inc., San Jose, CA, USA). Data are presented as means ± standard error (SEM). A *P* < 0.05 was determined to be statistically significant.

#### Microbiota Analyses

Chao1 rarefaction diversity, a measure of α-diversity, and UniFrac distance, a measure of β diversity, were calculated at baseline between groups and week 5 of the diet intervention for the PD group. Proportions of phyla (all confidence reads), families (92% confidence), and genera (95% confidence) representing %relative abundance were analyzed using OTUs with 98% similarity. Differences in proportions at baseline (PD and control group) and changes after diet intervention (PD group only) showing the presence/absence of specific OTUs with 95% similarity were calculated using the z-test (z score >1.96 or <-1.96 indicated significance of *P* < 0.05). Heat maps were generated to include all OTUs that reached significance. For qPCR assays, genome equivalents were used as the unit of comparison in an attempt to correct for differences in genome size and copy numbers of 16S rRNA genes. Paired *t*-tests between proportions of total DNA for fecal taxa were compared between PD and their matched control at baseline and week 5 of the diet intervention (PD group only) with a *P* < 0.05 determined to be statistically significant. If normality failed, the Wilcoxon Signed Rank test was used. Due to the exploratory nature of our study and the small sample size, *P*-values were not corrected for multiple comparisons.

## Results

### Participant Characteristics

A total of 16 participants (*n* = 8 PD group; *n* = 8 control group) agreed to participate in the study. All participants with PD completed the 2-week baseline and 5-week intervention period (Supplementary Figure 4). All participants in the control group provided urine and stool samples at baseline only. The majority of participants with PD were white males with a H&Y stage 2 on dopaminergic medication (Table 1). No statistical differences at baseline were observed in the PD group compared to controls for age (*P* = 0.95) or body mass index (BMI, *P* = 0.53).

**Table 1:** Baseline characteristics of participants in the Parkinson's disease and control groups^a^.

**Characteristics**	**Parkinson's disease (*n* = 8)**	**Controls (*n* = 8)**
Age (y)	71.4 ± 2.6	71.3 ± 2.4
Male participants, n (%)	5 (63)	3 (38)
Race/Ethnicity, n (%)		
White	8 (100)	7 (88)
American Indian or Alaska Native	-	1 (13)
Non-hispanic	7 (88)	8 (100)
BMI	26.7 ± 1.4	27.7 ± 0.7
Hoehn a Yahr Stage		
1	1 (13)	-
2	6 (75)	-
3	1 (13)	-
PD medications, n (%)		
L-DOPA	8 (100)	-
MAO-B inhibitors	3 (38)	-
MDS-UPDRS scores^b^		
Part I	8.9 ± 3.1	-
Part II	11.3 ± 2.9	-
Part III	31.9 ± 5.2	-
Part IV	2.6 ± 1.3	-
Total score	54.6 ± 9.9	-
MoCA score	26.6 ± 1.0	-

a *BMI, body mass index; L-DOPA, carbidopa-levodopa; MAO-B, monoamine oxidase B; MDS-UPDRS, Movement Disorder Society Unified Parkinson's Disease Rating Scale; MoCA, Montreal Cognitive Assessment. Values are represented as mean ± standard error of the mean; categorical variables are represented as total counts*.

b *Three participants were noted to be in the “OFF” period despite taking their Parkinson's medication*.

### Mediterranean Diet Adherence and Dietary Intake

Mediterranean diet adherence scores significantly increased after the 5-week diet intervention in the PD group (4.4 ± 0.6 vs. 11.9 ± 0.7; *P* < 0.01), indicating good adherence. All participants with PD (*n* = 8) completed at least 3 out of 4 scheduled telephone calls with study dietitians. Average dietary intake was compared before and after the Mediterranean diet intervention (Supplementary Table 4). Carbohydrate density (% total kcal), dietary fiber, total fat, total fat density, monounsaturated fat (including oleic acid) and polyunsaturated fat (including linoleic acid) significantly increased after the Mediterranean diet while cholesterol intake significantly decreased. Despite no significant changes in total energy intake after the diet, body weight decreased from 77.4 ± 5.4 kg to 74.9 ± 5.4 kg (*P* < 0.01).

### Gastrointestinal Function

Participants in the PD group reported significantly lower GSRS scores for constipation and indigestion symptoms during the final week of the intervention period (Table 2). Number of bowel movements per week (6.2 ± 0.9 vs. 7.5 ± 0.9) and average weekly stool form using the Bristol Stool Scale (3.0 ± 0.3 vs. 3.2 ± 0.3) were not significantly different after the diet intervention compared to baseline (*P* = 0.31 and *P* = 0.37, respectively).

**Table 2:** Gastrointestinal symptoms at baseline and after a 5-week Mediterranean diet intervention^a^.

**GSRS syndrome scores^b^**	**Parkinson's disease (*****n*** **=** **8)**		***P*-value**
	**Baseline**	**Intervention**	
Abdominal pain syndrome	1.52 ± 0.32	1.13 ± 0.06	0.13^c^
Diarrhea syndrome	1.10 ± 0.08	1.04 ± 0.04	1.00^c^
Constipation syndrome	2.25 ± 0.48	1.54 ± 0.31	0.04^*^
Indigestion syndrome	1.69 ± 0.21	1.41 ± 0.15	0.02^*^
Reflux syndrome	1.31 ± 0.25	1.06 ± 0.06	0.50^c^

a *GSRS, Gastrointestinal Symptom Rating Scale. Baseline vs. Intervention for the PD group was also compared using a paired t-test, unless otherwise indicated*.

* *P < 0.05*.

b *Symptoms for each syndrome are scored on a rating scale of 1 = no discomfort at all to 7 = very severe discomfort. Abdominal pain symptoms included abdominal pain, hunger pains, and nausea. Diarrhea symptoms included diarrhea, loose stools, and urgent need to defecate. Constipation symptoms included constipation, hard stools, and feeling of incomplete evacuation. Indigestion symptoms included rumbling, bloating, burping and gas. Reflux symptoms included heartburn and acid regurgitation*.

c *Compared using the non-parametric Wilcoxon Signed-Rank test*.

*Bolded values indicate significance*.

### Intestinal Permeability

Urinary excretion of sugars and ratios as measures of intestinal permeability were compared at baseline between control and PD groups and after a 5-week Mediterranean diet intervention for the PD group (Table 3). At baseline, only 5–24-h sucralose excretion was significantly lower in the PD group compared to controls. In the PD group, 0–5-h lactulose, 0–5-h erythritol, and 5–24-h sucralose excretion were significantly increased after the Mediterranean diet intervention. All other sugars and ratios for intestinal permeability were not significantly different.

**Table 3:** Markers of intestinal permeability at baseline and after a 5-week Mediterranean diet intervention^a^.

**Urinary sugar excretion (μmol)**	**Controls (*n* = 8)**	**Parkinson's disease (*****n*** **=** **8)**		***P*-value** **(PD vs. Control)**	***P*-value** **(Baseline vs. Intervention)**
		**Baseline**	**Intervention**		
0–5-h sucrose	9.7 ± 2.9	11.5 ± 4.7	28.2 ± 18.2	0.73	0.38^b^
0–5-h lactulose	16.8 ± 2.6	12.2 ± 1.6	21.4 ± 4.7	0.26	**0.02** ^*^ ^b^
0–5-h erythritol	3018 ± 315	2691 ± 296	3426 ± 289	0.52	**0.04** ^*^ ^b^
0–5-h L/E ratio	0.006 ± 0.001	0.005 ± 0.001	0.006 ± 0.001	0.64	0.49
5–24 h sucralose	51.6 ± 4.5	33.3 ± 5.9	47.0 ± 9.5	0.03^*^	**0.02** ^*^ ^b^
5–24 h erythritol	5915 ± 2484	2698.22 ± 197	3362 ± 636	0.15^b^	0.23
5–24 h S/E ratio	0.016 ± 0.003	0.012 ± 0.002	0.015 ± 0.002	0.14	0.25^b^
0–24 h sucralose	82.4 ± 7.3	65.5 ± 11.1	81.7 ± 15.6	0.31	0.15^b^
0–24 h erythritol	8933 ± 2638	5389 ± 442	6788 ± 893	0.15^b^	0.08
0–24 h S/E ratio	0.013 ± 0.002	0.012 ± 0.001	0.011 ± 0.001	0.61	0.59

a *PD, Parkinson's disease; L/E, lactulose/erythritol; S/E, sucralose/erythritol. Values represent mean ± standard error of the mean. Participants with PD at baseline were matched with controls and compared using paired t-test. Baseline vs. Intervention for the PD group was also compared using a paired t-test, unless otherwise indicated*.

* *P < 0.05*.

b *Compared using the non-parametric Wilcoxon Signed-Rank test*.

*Bolded values indicate significance*.

### Microbial Communities

After removal of low-quality reads (those with short-read length or with low-quality score), a total of 438,192 sequences (mean of 18,258 sequences per sample, with an average length of 315 nucleotides per read) were retained. A total of 1607 OTUs and 1580 OTUs were retained at similarity level of 98% and 95% respectively. The α- and β-diversity indexes used to measure within sample richness and population diversity did not differ between control or PD groups nor change after the diet intervention (Supplementary Figures 5–7).

OTUs with 98% similarity were used to compare proportions of taxa. While the proportion of Proteobacteria was not different between PD and control groups at baseline (4.5 ± 1.3% vs. 3.0 ± 0.7%; *P* = 0.80), it was significantly increased after the intervention (5.8 ± 1.6%; *P* = 0.01). The proportion of *Desulovibrionaceae* (phylum Proteobacteria) was significantly higher at baseline in the PD group compared to controls (1.1 ± 0.2% vs. 0.3 ± 0.1%; *P* < 0.01) and reduced at week 5 of the diet intervention (0.9 ± 0.2%; *P* = 0.04). This may be explained by the proportion of *Bilophila*, which was also elevated at baseline (0.6 ± 0.1% vs. 0.2 ± 0.1%; *P* < 0.01) and decreased in the PD group after the diet intervention (0.5 ± 0.1%; *P* = 0.01). In addition, the proportion of *Roseburia* (phylum Firmicutes) was significantly lower in PD compared to controls (0.6% ± 0.2 vs. 1.6% ± 0.3; *P* = 0.03) and increased at week 5 (0.9 ± 0.2%; *P* < 0.01). All other proportions of taxa were not different at baseline nor changed after the diet intervention. The two phyla, Bacteriodetes and Firmicutes dominated in all samples. At baseline, the proportion of Firmicutes was significantly lower in PD compared to controls (42.8 ± 3.1% vs. 53.1 ± 2.1%; *P* = 0.04) and was unchanged after the diet intervention (40.8 ± 1.8%; *P* = 0.34).

**Figure 1:**
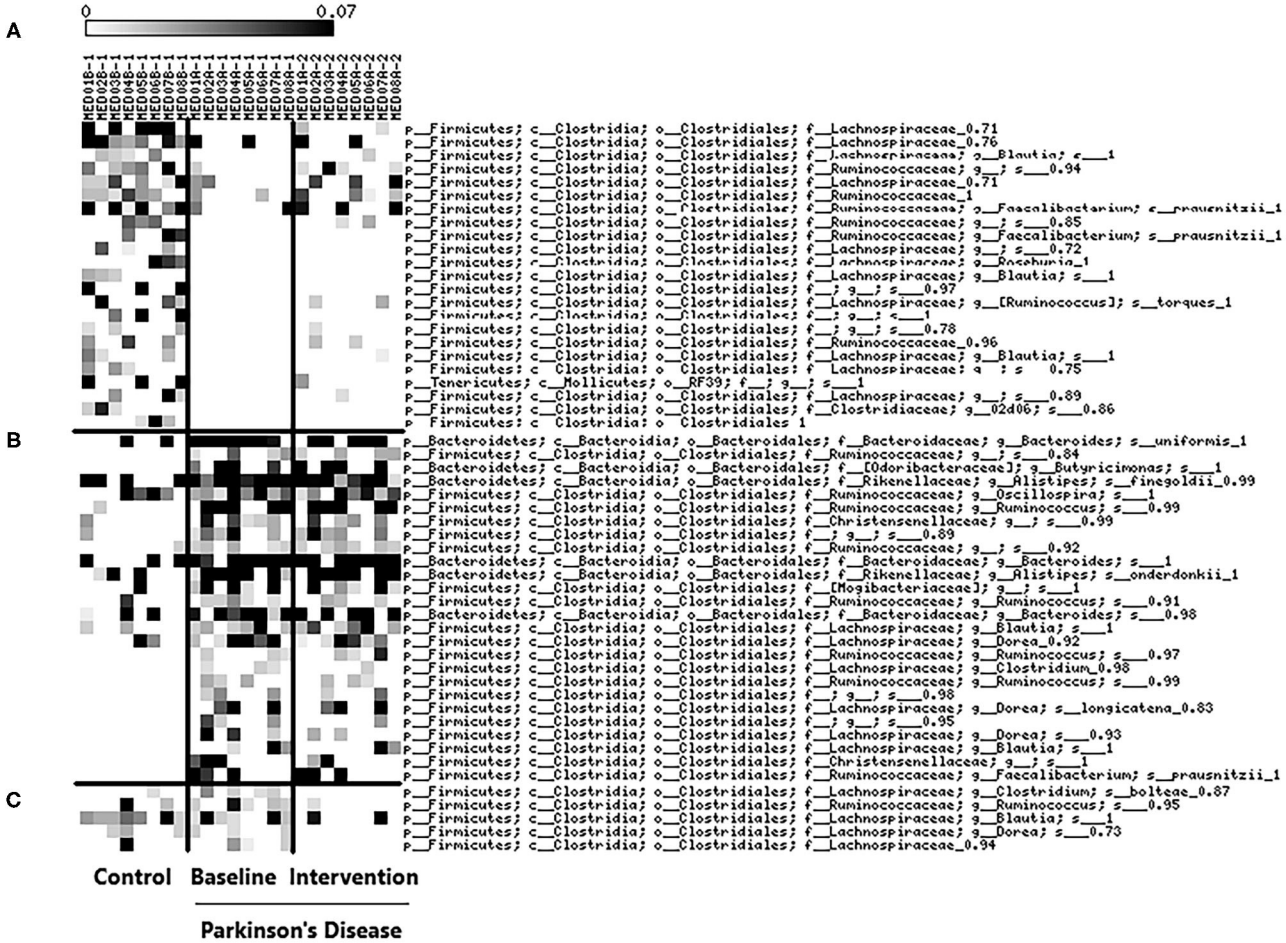
Heat-map of prevalent bacterial OTUs (determined by z-score) with 95% similarity at baseline between control (*n* = 8) and PD groups (*n* = 8) and changes after a 5-week Mediterranean diet intervention (PD group; *n* = 8). **(A)** OTUs that were more prevalent in the control group compared to PD group at baseline. **(B)** OTUs that were more prevalent in the PD group compared to control group at baseline. **(C)** OTUs that decreased after the Mediterranean diet intervention in the PD group.

We identified OTUs with 95% similarity to determine individual OTUs that were prevalent at baseline (control and PD group) and after the Mediterranean diet intervention (Figure 1). At baseline, multiple OTUs for *Lachnospiraceae, Ruminococcaceae, Roseburia, Blautia*, and *F. prausnitzii* were less prevalent in the PD group compared to controls. OTUs that were identified to be more prevalent in the PD group included: *Christensenellaceae, Butyricimonas, Oscllospira*, and *Dorea*. Interestingly, multiple OTUs for *Blautia* were also shown to be more prevalent in the PD group at baseline. OTUs corresponding to *Clostridium bolteae, Ruminococous, Blautia, Dorea* and *Lachnospiraceae* decreased in prevalence in the PD group after the diet intervention suggesting short-term adherence to a Mediterranean diet may mediate changes in intestinal microbiota profiles. The targeted qPCR analysis did not show differences between groups at baseline nor changes after the diet intervention in quantities of bifidobacteria, LAB, *E.coli, A. municiphila, Prevotella*, and *F. prausintizi* (Supplementary Table 5).

## Discussion

The primary aim of this study was to test the feasibility of a Mediterranean diet intervention and its impact on GI function, intestinal permeability and fecal microbial communities in PD patients. Adherence to a Mediterranean diet was shown to be feasible in PD patients as demonstrated by an increase in MEDAS scores (≥10 indicating “good adherence”) at the end of the 5-week diet intervention. MEDAS scores have been validated against 3-day food records as an accurate and reliable assessment of Mediterranean diet adherence (24). Dietary changes likely were re-enforced by participant's compliance with weekly telephone follow ups as telephone interactions have previously been shown to promote dietary behavior change (39). We also observed unintended loss of body weight after the intervention. It is possible weight loss was attributed to decreased total energy intake, however we did not observe this when measuring energy intake by 24-h dietary recall. Our dietary assessment might have lacked accuracy as under- and/or overestimate of food intake has been reported (40). The Mediterranean diet is associated with promotion of healthy weight loss in overweight and obese individuals (41), which was also seen in our study. In PD, individuals with lower BMI are associated with higher UPDRS scores and increased risk of malnutrition (42, 43). Therefore, weight status should be considered before making dietary changes and future interventions should characterize diet effects on body composition and nutritional status in this population.

GSRS constipation syndrome and indigestion scores significantly decreased from slight discomfort to no discomfort after the diet intervention in participants with PD suggesting the Mediterranean diet may be an effective intervention for reducing GI symptoms in this population. This may be attributed to the increased intake of dietary fiber observed in this study as this is in line with recommendations for treatment of functional constipation (44). Limited studies have evaluated the effectiveness of diet interventions on constipation symptoms in PD. Recently, one study demonstrated increased stool frequency and softer stool form as well as reduction in laxative usage using synbiotic fermented milk containing 7.8 g of fiber in people with PD (45). Usage of fiber and probiotic supplements in this population have also been documented in smaller studies (46). Optimal management of constipation symptoms in PD has yet to be determined and findings from this study could support the usage of Mediterranean diet recommendations in this population.

Urinary excretion ratios of markers of intestinal permeability did not differ at baseline between groups, nor changed after the diet intervention. This is in contrast with earlier findings which demonstrated PD is associated with increased lactulose/mannitol ratio (small intestine permeability) (47). Further, a previous study has documented increased 24-h sucralose excretion in PD compared to healthy controls (8). While 24-h sucralose excretion was not different at baseline in this study, we did observe lower excretion of the 5–24-h sucralose at baseline in the PD group compared to controls and a significant increase after the 5-week Mediterranean diet intervention. Single sugar excretion for 0–5-h lactulose and erythritol were also increased after the diet intervention. It's possible these changes were observed due to faster intestinal transit and increased exposure to absorptive surface area of the enterocytes. Differences observed between studies may be attributed to small sample sizes (*n* < 20) and methodological differences for administering and measuring sugar probes as permeability markers. Newer methods used in this study have been validated by van Wilk et al. to use site-specific timing (small intestine [0–5 h], colonic [5–24 h], and whole gut [0–24 h]), lower dosage of lactulose (1 g vs. 7.5 g) and sugars of smaller molecular weight (erythritol) to normalize for transit time and kidney function (28).

Though microbiota diversity was not different at baseline between groups or after the diet intervention, differences in proportions of genera were observed between groups at baseline and after the 5-week Mediterranean diet intervention. Specifically, proportions for *Roseburia* decreased and *Bilophila* was increased at baseline. *Roseburia* spp. are butyrate-producing members of Firmicutes and have been shown to be reduced in inflammatory bowel diseases (48, 49) and PD in a recent meta-analysis (50). Interestingly, *Roseburia* spp. may be involved in immune system regulation (51) and are considered primary degraders of dietary β-mannans (i.e., found in foods such as nuts, tomatoes, and legumes) that respond rapidly to supplementation *in vivo* (52). This may explain increases in proportion of *Roseburia* observed after the Mediterranean diet intervention. *Bilophila* is sulfite-reducing member of Proteobacteria that can produce hydrogen sulfide which may impair colonocyte function and butyrate oxidation (53). Other studies have also shown this genus to be increased in PD, correlated with H&Y staging and involved in regulation mucin-degraders like *Akkermansia municiphila* (54, 55). Further, taxonomic differences in *Roseburia, Bilophila, Akkermansia, Prevotella, Faecalibacterium, Dorea, Blautia*, and others have been correlated with constipation symptoms in PD (56). This may explain changes in prevalence observed in these taxa after the Mediterranean diet intervention, but these observations should be confirmed in future studies.

The L-rhamnose mispackaging was an unforeseen limitation of the study as it was originally intended to control for the ratio of upper GI permeability. However, our use of erythritol also met the requirements as it is rapidly absorbed by the small intestine, contains a similar molecular weight and less likely to be fermented by bacteria (57). Our study was limited by the lack of a control group during the intervention period and small sample size which reduced our power to observe differences in intestinal permeability and microbiota. Furthermore, because this was a small feasibility study to generate future hypotheses, results, including weight loss after the Mediterranean diet intervention, should be interpreted with caution.

## Conclusion

In this preliminary study, we demonstrated that short-term adherence to a Mediterranean diet is feasible and may be effective in improving constipation symptoms and modulating microbiota but not intestinal permeability in people with PD. Future studies should be conducted to confirm the effect of a Mediterranean diet intervention on constipation symptoms, gut microbiota and intestinal permeability in a larger cohort.

## Data Availability Statement

The datasets presented in this study can be found in online repositories. The names of the repository/repositories and accession number(s) can be found at: PRJNA780943.

## Ethics Statement

The studies involving human participants were reviewed and approved by Institutional Review Board-01 of the University of Florida. The patients/participants provided their written informed consent to participate in this study.

## Author Contributions

CR, MB, VM, and BL-H designed the research. CR, MB, LT, CN, and BL-H were responsible for the recruitment, coordination, and data collection of participants. CR and MB conducted the diet education and weekly follow up with participants. SC, BP, and AR-Z conducted the neurological evaluations. CR, JS, and YW conducted urine analyses, CR, MU, MT, and VM conducted stool analyses and the interpretation. CR, MU, and BL-H performed the statistical analysis. CR, MU, VM, JS, YW, and BL-H wrote the manuscript. All authors read and approved the final manuscript.

## Funding

This project was funded as part of the University of Florida's Creating the Healthiest Generation Moonshot initiative, which was supported by the UF Office of the Provost, UF Office of Research, UF Health, UF College of Medicine, and the UF Clinical and Translational Science Institute as well as the USDA National Institute of Food and Agriculture, Hatch project FLA-FOS-005636 and the Lauren and Lee Fixel Family Foundation.

## Conflict of Interest

The authors declare that the research was conducted in the absence of any commercial or financial relationships that could be construed as a potential conflict of interest.

## Publisher's Note

All claims expressed in this article are solely those of the authors and do not necessarily represent those of their affiliated organizations, or those of the publisher, the editors and the reviewers. Any product that may be evaluated in this article, or claim that may be made by its manufacturer, is not guaranteed or endorsed by the publisher.
