# Supplementary material for: Mediterranean Diet Adherence in People With Parkinson's Disease Reduces Constipation Symptoms and Changes Fecal Microbiota After a 5-Week Single-Arm Pilot Study
Source: Front Neurol. 2021 Dec 23;12:794640. doi: 10.3389/fneur.2021.794640 (PMC8733603; doi:10.3389/fneur.2021.794640)
Supplement: Supplementary file 1 [file Data_Sheet_1.docx]

**SUPPLEMENTARY METHODS AND RESULTS**

Carley Rusch^1,2^*, Matthew Beke^1,2^, Lily Tucciarone^1^, Carmelo Nieves Jr.^1^, Maria Ukhanova^3^, Massimiliano S. Tagliamonte^4^, Volker Mai^3^, Joon Hyuk Suh^5^, Yu Wang^5^, Shannon Chiu^2^, Bhavana Patel^2^, Adolfo Ramirez-Zamora^2^, Bobbi Langkamp-Henken^1^

^1^ Food Science and Human Nutrition Department, University of Florida, Gainesville, FL, United States

^2^ Norman Fixel Institute for Neurological Diseases, Department of Neurology, University of Florida, Gainesville, FL, United States

^3^ Emerging Pathogens Institute, Department of Epidemiology, University of Florida, Gainesville, FL, United States

^4^ Emerging Pathogens Institute, Department of Pathology, Immunology and Laboratory Medicine, University of Florida, Gainesville, FL, United States

^5^ Food Science and Human Nutrition Department, Citrus Research and Education Center University of Florida, Lake Alfred, FL, United States

***Corresponding author**: Carley Rusch; [crusch792@ufl.edu](mailto:crusch792@ufl.edu)

**METHODS**

**Participant Inclusion/Exclusion Criteria**

Participants with Parkinson’s disease (PD) and their healthy partners (controls) were included in the study if they 1) were between 21-85 years old (inclusive), 2) currently lived in the same household with an eligible partner who is willing to participate, 3) were able to complete informed consent in English, 4) willing to avoid beer, wine, and cocktails on the day before and the day of the study visits, 5) willing to fast (no food or drink, except water or tea) for the first 5 hours of the study urine collections, 6) willing to maintain usual diet through the pre-baseline period, 7) willing to discontinue taking prebiotic, fiber, probiotic, herbal, or high-dose vitamin or mineral supplements throughout the study protocol, 8) willing to limit use of oral laxative medication to an “as-needed basis” (i.e. <3 times per week) during the study protocol, 9) willing to avoid high intensity exercises two days prior to and the day of the permeability tests, 10) had not used non-steroidal anti-inflammatory drugs (NSAIDs) daily in the past 3 months or incidental use in the last 2 weeks prior to screening, 11) had not used anticholinergic, prokinetc agents, enemas, or suppositories, 12) had not used another investigational product within 3 months of screening, 13) had not used antibiotics within 2 months from day of stool collection, 14) did not score >6 on the Mediterranean diet adherence screener (MEDAS) (1, 2), and 15) had not been diagnosed with a GI disease or condition.

Additional criteria for participants with PD included diagnosis of PD according to the MDS Clinical Diagnostic Criteria by a movement disorders trained neurologist (3), Hoehn & Yahr (H&Y) stage ≤3, willing to make dietary changes to follow a Mediterranean dietary pattern during the intervention period, had not been diagnosed with atypical or secondary Parkinsonism, and had no history of deep brain stimulation surgery. Partners were included if they did not have history of a neurological disease or condition, diabetes, or liver disease.

**Intestinal Permeability LC-MS/MS analysis**

*Preparation of internal standards and calibration solutions*

The analytes including sucralose, erythritol, sucrose, L-rhamnose and lactulose were purchased from Sigma-Aldrich (St. Louis, MO, USA). For the internal standards, sucralose-d_6_ was obtained from Cayman Chemical (Ann Arbor, MI, USA), [UL-^13^C_4_]erythritol and [UL-^13^C_12_]lactulose were purchased from Omicron Biochemical Inc (South Bends, IN, USA), and [UL-^13^C_12_]sucrose was purchased from Santa Cruz Biotechnology (Santa Cruz, CA, USA). Stock solutions of each analyte and internal standard were prepared at a concentration of 1,000 μg/mL in methanol and stored at -80°C until analyses. Standard mixture solutions for calibration curves were prepared daily by diluting stock solutions with 90% acetonitrile solution (10/90, v/v). The ranges of calibration standards were 0.05-20 μg/mL for sucralose, L-rhamnose and lactulose, and 0.25-100 μg/mL for erythritol.

*Urine sample preparation*

Urine samples were extracted by aliquoting 20 μL of urine into 1.5 mL plastic tube and mixed with 175 μL of 90% acetonitrile solution (10/90, v/v) and 5 μL of internal standard mixtures (concentration: 200 μg/mL) to a total volume of 200 μL. The sample was vigorously vortexed for 20 min and centrifuged at 20000 *xg* for 10 min at 4°C. The supernatant was transferred into a vial and 5 μL of this solution was injected into LC–MS/MS system. The extraction of samples was performed in triplicate.

*Optimized LC–MS/MS procedure*

Analytes (sucralose, erythritol, sucrose, and lactulose) were separated on a Supelco TSKgel Amide-80 column (2.0 × 150 mm, particle size 3.0 μm) at a column temperature of 40°C using a gradient elution with 10 mM ammonium acetate as eluent A and acetonitrile as eluent B (Fisher Scientific, Fair Lawn, NJ, USA). The gradient was as follows: 0-14 min 90-70% B, 14-16 min 70-50% B and 16-19 min 50% B. The column was re-equilibrated in 7 min using the initial composition of mobile phase. The flow rate was set at 0.2 mL/min. The mass spectrometer was equipped with an electrospray ionization (ESI) interface, operating in the negative ionization mode. The ESI parameters were as follows: spray voltage, 2500 V; ion transfer tube temperature, 325°C; vaporizer temperature, 275°C; sheath gas, 35 Arb; aux gas, 10 Arb; and sweep gas, 0 Arb. The MS/MS detection was operated using selective reaction monitoring (SRM) mode. Dwell time was 100 msec, and CID gas was set at 2 mTorr. MS/MS parameters for each analyte were optimized using flow injection analysis of individual standards. The optimum values including a SRM transition for quantification, collision energies and RF lens are shown in **Supplementary Table 1**. Xcalibur software (Ver. 2.2) was used for data processing and instrument control. Calibration curves were constructed by plotting the peak area ratio of analyte to internal standard against the nominal analyte concentration with an acceptable coefficient of variation of ≤ 15%.

**Supplementary Table 1.** Optimal MS/MS parameters including SRM transition, collision energy and RF lens for the analytes and internal standards (IS).

| **Analyte** | ***R*_t_ (min)** | **Polarity** | **Q1 (m/z)** | **Q3 (m/z)** | **CE (V)** | **RF lens (V)** |
| --- | --- | --- | --- | --- | --- | --- |
| sucralose | 4.1 | negative | 397.1 | 361.0 | 12 | 74 |
| sucralose-d_6_ (IS) | 4.1 | negative | 403.1 | 367.1 | 12 | 74 |
| erythritol | 7.6 | negative | 121.1 | 89.1 | 10 | 39 |
| [UL-^13^C_4_]erythritol (IS) | 7.6 | negative | 125.1 | 92.2 | 10 | 39 |
| sucrose | 15.7 | negative | 341.1 | 179.1 | 14 | 66 |
| [UL-^13^C_12_]sucrose (IS) | 15.7 | negative | 353.2 | 123.2 | 14 | 66 |
| lactulose | 16.7 | negative | 341.1 | 161.0 | 10 | 50 |
| [UL-^13^C_12_]lactulose (IS) | 16.7 | negative | 353.2 | 167.1 | 10 | 50 |

*Purity assay*

The purity of sugars (sucralose, erythritol, sucrose, lactulose and L-rhamnose) were verified by LC–MS with full scan (FS) mode (m/z 100-500). The purity was determined as the percentage of peak area of the sugar relative to the total peak area in the chromatogram.

*Unknown impurity assay*

For the product labeled “L-rhamnose” during permeability testing, additional identification processes were conducted using LC–HRMS (Q-Exactive Plus Orbitrap, Thermo Fisher Scientific, San Jose, CA, USA) to confirm an unknown impurity in the product. The mass spectrometer was equipped with a heated electrospray ionization (HESI-II) interface, operating in full scan (FS), targeted-selected ion monitoring (t-SIM) and t-SIM/data-dependent MS2 (t-SIM/ddMS2) negative ionization modes. The FS was performed with a mass range from m/z 100 to m/z 500. t-SIM and t-SIM/ddMS2 were used for the targeted monitoring of L-rhamnose and the impurity in MS1 and MS/MS levels, respectively. The scans were acquired at a resolution of 70,000 for MS1 and 17,500 for MS/MS. The HESI-II parameters were as followed: spray voltage, 2.5 kV; capillary temperature, 275°C; aux gas heater temperature, 300°C; sheath gas, 35 psi; aux gas, 10 psi; sweep gas, 1 psi; and S-lens RF level, 50%.

**Targeted quantitative polymerase chain reaction (qPCR) analysis**

qPCR analysis was performed in duplicate using a PowerUp SYBR Green master Mix (ThermoFisher) on a CFX-Connect RealTime System (BioRad). Reactions were performed in a final volume of 12.5 µl with10 ng DNA template and 0.2 µM of each primer (Invitrogen). The following primers and annealing temperatures were used: 1) bifidobacteria (F: 5’-TCGCGTC(C/T)GGTGTGAAAG-3’; R: 5’-CCACATCCAGC(A/G)TCCAC-3’, 58°C) and eubacteria (V3) (F: 5'-CCTACGGGAGGCAGCAG-3'; R: 5'-ATTACCGCGGCTGCTGG-3', 56°C) representing *Bifidobacteria* spp.; 2) lactic acid bacteria (LAB) (F: 5’-AGCAGTAGGGAATCTTCCA-3’; R: 5’-ATTYCACCGCTACACATG-3’, 58°C), 3) *Escherichia coli* (F: 5’-CAATTTTCGTGTCCCCTTCG-3’; R: 5’-GTTAATGATAGTGTGTCGAAAC-3’, 57°C), 4) *Akkermansia muciniphila* (F: 5’-CAGCACGTGAAGGTGGGGAC-3’; R: 5’-CCTTGCGGTTGGCTTCAGAT-3’, 60°C), 5) *Prevotella* (F: 5’-CCAGCCAAGTAGCG TGCA-3’, R: 5’- TGGACCTTCCGTATTA CCGC-3’, 56°C), 6) *Faecalibacterium prausitzii* (F: 5’-AGATGGCCTCGCGTCCGA-3’, R: 5’-CCGAAGACCTTCTTCCTCC-3’, 60°C).

**RESULTS**

**Purity of sugars**

The purity of sugars (sucralose, erythritol, sucrose, lactulose and L-rhamnose) used in permeability testing were evaluated (**Supplementary Table 2**). The results indicated that the purity was higher than 89.9% for the tested sugars except for L-rhamnose. The product labeled “L-rhamnose” did not actually contain L-rhamnose, but had a major unknown impurity observed (>90.0% of total impurities) at m/z 377 on the chromatogram. Due to this issue, L-rhamnose was excluded from the sugar monitoring in the urine samples.

**Supplementary Table 2.** Purity of sugars used in permeability testing

| Analyte | Labeled manufacturer purity (amount) | Measured purity (amount) |
| --- | --- | --- |
| sucralose | N/A | 90.3% |
| erythritol | N/A | 96.2% |
| sucrose | N/A | 95.8% |
| lactulose | 100.0% (10 g/15 mL) | 89.9% (8.99 g/15 mL) |
| L-rhamnose | N/A | 0.0% |

**Identification of an unknown impurity in “L-rhamnose”**

The major impurity was observed at m/z 377.2082 ([M - H]^-^) on the FS chromatogram and the corresponding molecular formula (C_20_H_30_N_2_O_5_) was calculated. Next, t-SIM/ddMS2 was employed to gather the information on product ions fragmented from the precursor ion (m/z 377.2082). Three representative product ions were observed at m/z 200.0717, 301.1922 and 345.1816 on the MS/MS spectrum. An artificial sweetener, neotame, was selected as the candidate of the impurity based on the collected information and previous literature (4, 5). Finally, to fully confirm the impurity of the L-rhamnose product, the authentic standards of L-rhamnose and neotame (the candidate impurity) were analyzed in t-SIM and t-SIM/ddMS2 modes. As seen in **Supplementary Figure 1**, the L-rhamnose product only contained neotame (m/z 377.2082) with no L-rhamnose (m/z 163.0612) observed. The pattern of product ions from the L-rhamnose product coincided with that from the neotame standard (**Supplementary Figure 2**). Taken together, the results indicated that the product labeled “L-rhamnose” was indeed neotame.

**Monitoring of sugars in urine samples**

The LC–MS/MS chromatograms of the analytes and internal standards are shown in **Supplementary Figure 3**. The analytes (sucralose, erythritol, sucrose and lactulose) were favorably separated on the HILIC-based amide stationary phase, without any visible interference around their retention times. The calibration curve for each analyte exhibited good linearity over the concentration range, with high correlation of determination values (*r*^2^ > 0.99) (**Supplementary Table 3**). The limit of quantification, which was set to ten times a signal-to-noise ratio, demonstrated high sensitivity to determine trace amounts of sugars.

**Supplementary Table 3.** Linearity and limit of quantification (LOQ) for the analytes.

| **Analyte** | **Calibration range (μg/mL)** | **Slope** | **Intercept** | ***r*^2^** | **LOQ** |
| --- | --- | --- | --- | --- | --- |
| sucralose | 0.05-20 | 0.2828 | 0.0010 | 0.9995 | 0.05 |
| erythritol | 0.25-100 | 0.2602 | -0.0792 | 0.9993 | 0.25 |
| sucrose | 0.05-20 | 0.7480 | 0.0140 | 0.9990 | 0.05 |
| lactulose | 0.05-20 | 0.0919 | 0.0039 | 0.9992 | 0.05 |


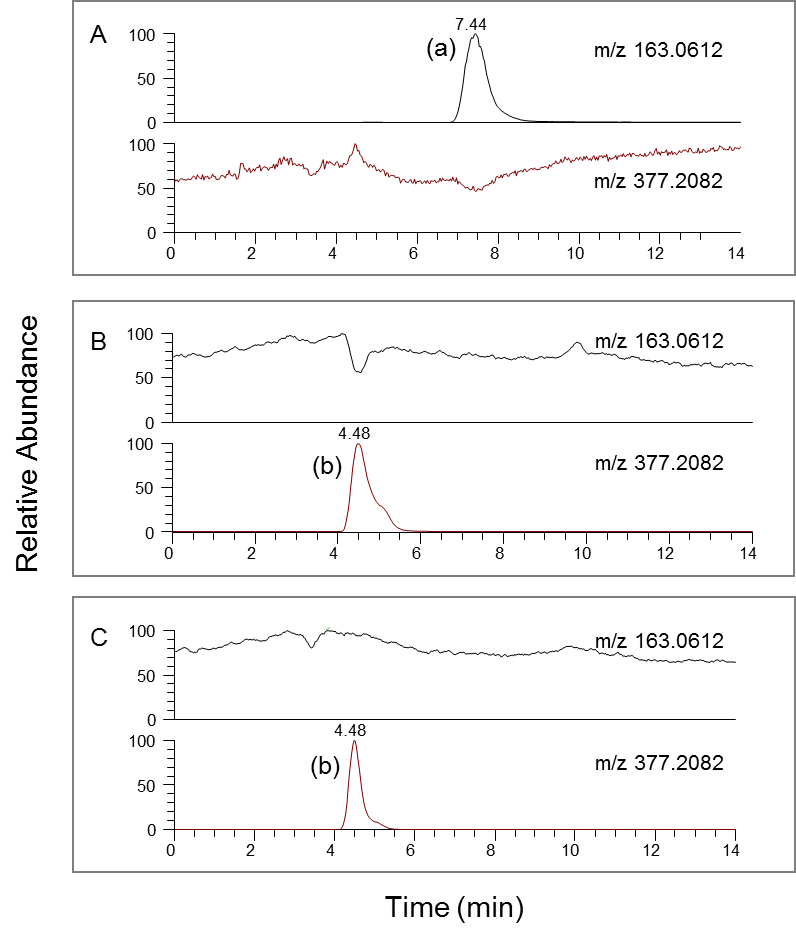


**Supplementary Figure 1.** HRMS t-SIM chromatograms of (A) L-rhamnose standard, (B) neotame standard, and (C) the product labeled “L-rhamnose” used in the permeability testing: L-rhamnose peak at m/z 163.0612 (a) and neotame peak at m/z 377.2082 (b).


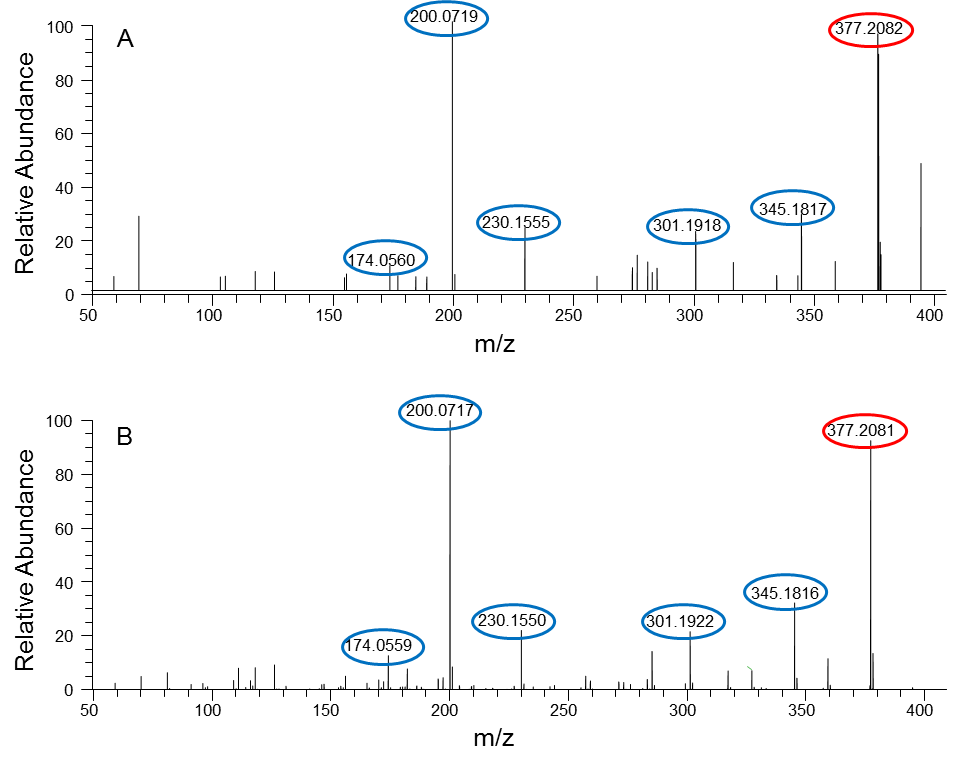


**Supplementary Figure 2.** HRMS t-SIM/ddMS2 spectra of (A) neotame standard, and (B) the product labeled “L-rhamnose” used in the permeability testing: red and blue marks indicate precursor and product ions, respectively.


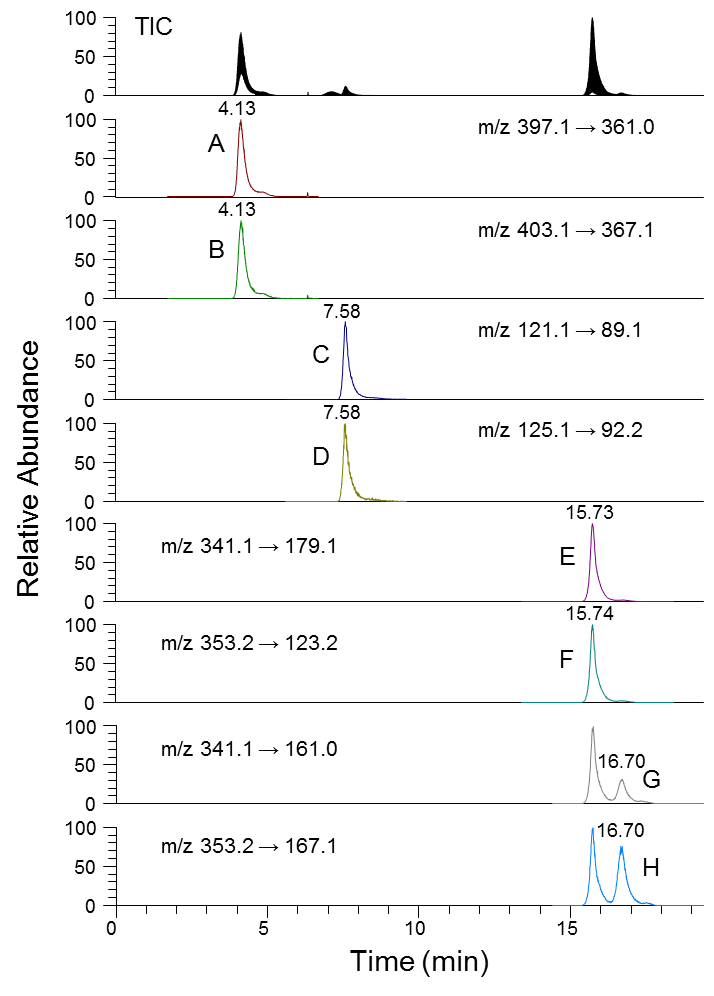


**Supplementary Figure 3.** LC–MS/MS SRM chromatograms of the analytes and internal standards (IS): (A) sucralose, (B) sucralose-d_6_ (IS), (C) erythritol, (D) [UL-^13^C_4_]erythritol (IS), (E) sucrose, (F) [UL-^13^C_12_]sucrose (IS), (G) lactulose, and (H) [UL-^13^C_12_]lactulose (IS).

**
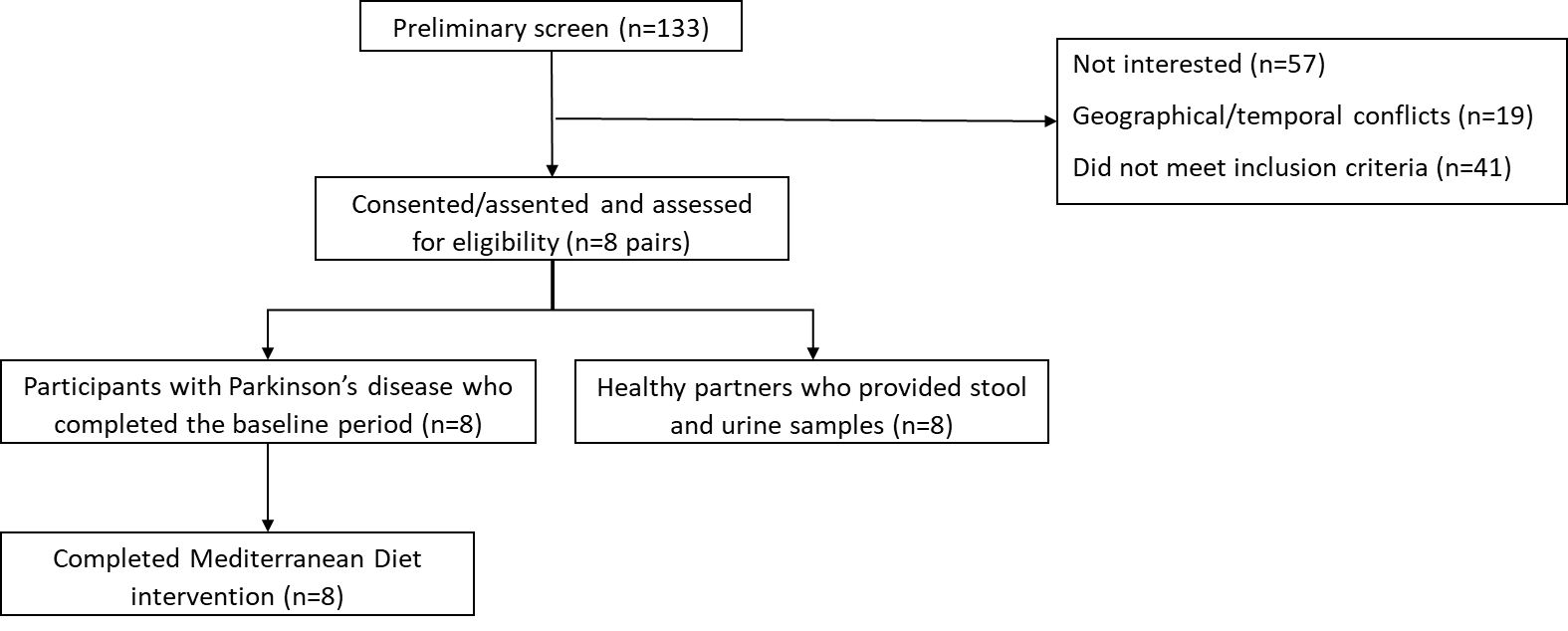
**

**Supplementary Figure 4.** Participant flow chart.

**Supplementary Table 4.** Average dietary intake at baseline and after a 5-week Mediterranean diet intervention.

| **Characteristics^a^** | **Parkinson’s disease (n=8)** | | ***P*-value** |
| --- | --- | --- | --- |
|  | **Baseline** | **Intervention** |  |
| Total kcal | 2255 ± 94 | 2578 ± 201 | 0.130 |
| Protein (g) | 84.3 ± 5.8 | 86.9 ± 6.4 | 0.738 |
| Protein (%kcal) | 15.0 ± 0.8 | 13.9 ± 1.2 | 0.535 |
| Carbohydrate (g) | 268.8 ± 19.8 | 287.5 ± 34.2 | 0.508 |
| Carbohydrate (%kcal) | 47.9 ± 3.3 | 44.5 ± 3.6 | **0.036*** |
| Dietary fiber (g) | 24.8 ± 2.1 | 36.2 ± 3.6 | **0.027*** |
| Sugar (g) | 138.2 ± 18.8 | 136.5 ± 26.4 | 0.938 |
| Total fat (g) | 95.4 ± 7.3 | 127.1 ± 15.1 | **0.035*** |
| Total fat (%kcal) | 37.8 ± 2.0 | 43.8 ± 3.4 | **0.040*** |
| Saturated (g) | 29.5 ± 3.0 | 28.7 ± 3.2 | 0.833 |
| Saturated (%kcal) | 5.2 ± 0.4 | 4.4 ± 0.3 | 0.166 |
| Monounsaturated (g) | 33.0 ± 2.5 | 56.2 ± 8.8 | **0.025*** |
| Monounsaturated (%kcal) | 5.8 ± 0.3 | 8.6 ± 1.1 | **0.020*** |
| 18:1 Oleic Acid (g) | 31.0 ± 2.4 | 53.5 ± 8.6 | **0.026*** |
| Polyunsaturated (g) | 25.2 ± 2.8 | 33.6 ± 3.7 | **0.009**** |
| Polyunsaturated (%kcal) | 4.5 ± 0.5 | 5.2 0.6 | 0.168 |
| 18:2 Linoleic Acid (g) | 21.9 ± 2.4 | 29.9 ± 3.3 | **0.006**** |
| 18:3 α-linolenic acid (g) | 2.7 ± 0.4 | 3.1 ± 0.6 | 0.439 |
| 20:5 EPA (mg) | 46.1 ± 20.3 | 93.3 ± 35.0 | 0.316 |
| 22:5 DHA (mg) | 133.0 ± 38.1 | 166.0 ± 54.2 | 0.647 |
| Cholesterol (mg) | 384.2 ± 60.3 | 296.1 ± 57.5 | **0.027*** |

^a^ kcal, kilocalorie; g, grams; EPA, eicosapentaenoic acid; DHA, docosahexaenoic acid. Values represent mean ± standard error of the mean. Average dietary intake represents average of 3, 24-hour recalls (2 weekday, 1 weekend) for each time point using the Automated Self-Administered 24-hour Dietary Assessment Tool, version 2018 (6). Baseline vs. Intervention for the PD group were compared using a paired t-test, unless otherwise indicated. **P*<0.05; ***P*<0.01

**Supplementary Figure 5.** Refraction curve (Observed OTUs) showing average amount of OTUs at baseline between control (n=8) and PD groups (n=8) and changes after a 5-week Mediterranean diet intervention (PD group; n=8) as a function of the number of sequences reads.


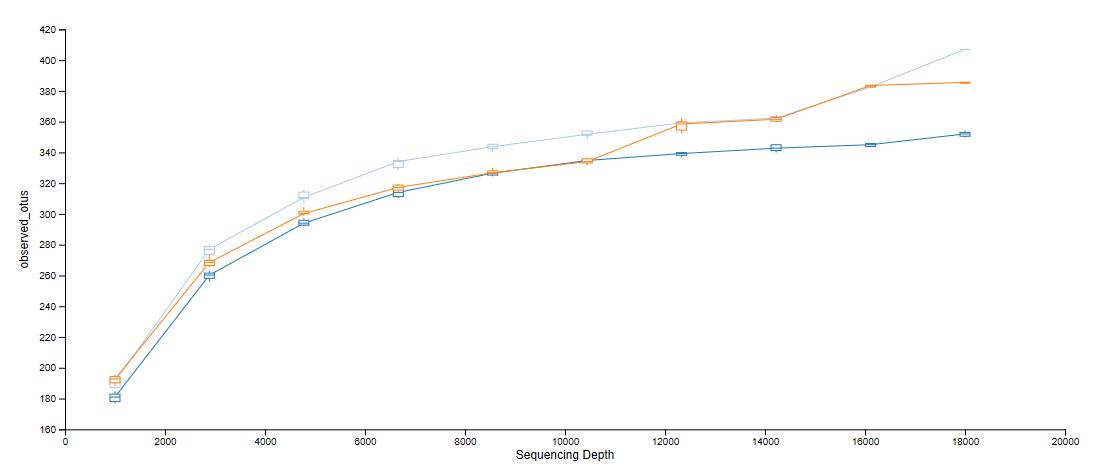


Observed OTUs


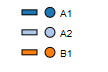

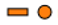


Control

Baseline

Intervention


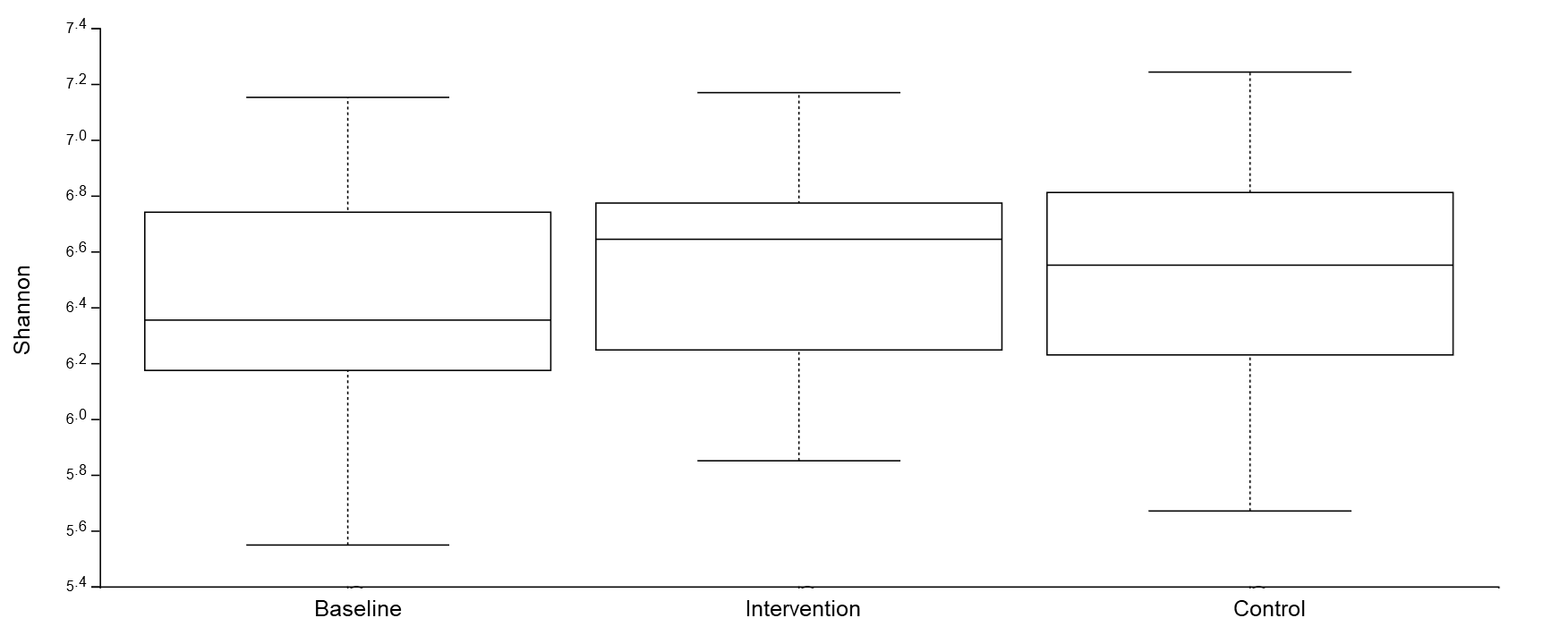
**Supplementary Figure 6.** Alpha diversity distributions of control (n=8) and PD groups (n=8) at baseline and changes after a 5-week Mediterranean diet intervention (PD group; n=8). No significant difference was found between groups.

**
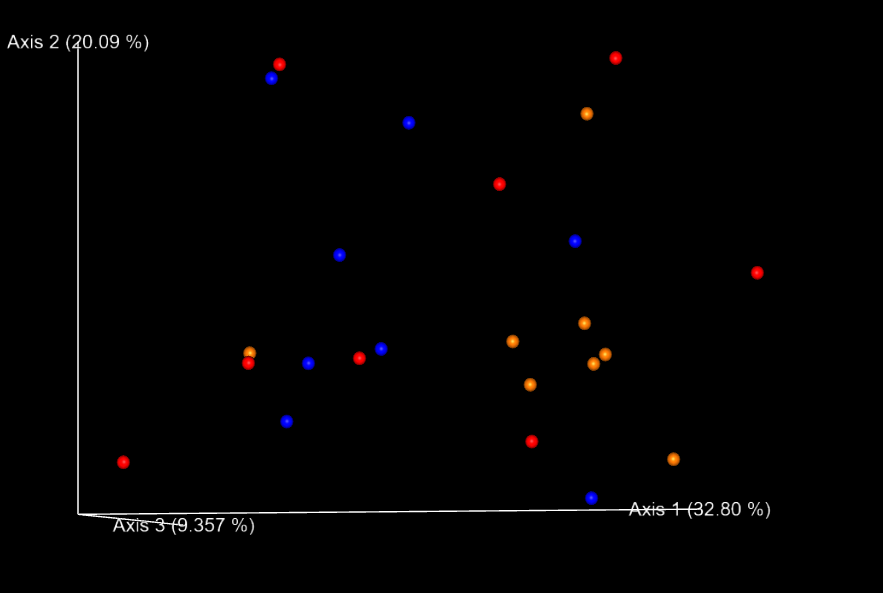
**

Baseline

Intervention

Control

**Supplementary Figure 7.** Principle coordinate plot based on weighted UniFrac distances (β-diversity) demonstrates microbiota composition in fecal samples measured at baseline between control (n=8) and PD groups (n=8) and changes after a 5-week Mediterranean diet intervention (PD group; n=8). Samples from each group are color coded. No significant difference in distances between groups was found.

**Supplementary Table 5.** qPCR analysis of selected fecal taxa at baseline and after a 5-week Mediterranean diet intervention^a^

| **Taxon (% total DNA)** | **Controls (n=8)** | **Parkinson’s disease (n=8)** | | ***P*-value**  **(PD vs. Control)** | ***P*-value**  **(Baseline vs. Intervention)** |
| --- | --- | --- | --- | --- | --- |
|  |  | **Baseline** | **Intervention** |  |  |
| Bifidobacteria | 0.31 ± 0.19 | 2.54 ± 1.93 | 1.31 ± 0.94 | 0.58^b^ | 0.22^b^ |
| Lactic acid bacteria | 0.16 ± 0.12 | 0.77 ± 0.71 | 0.58 ± 0.52 | 0.84^b^ | 0.74^b^ |
| *Escherichia coli* | 0.0041 ± 0.0008 | 0.0059 ± 0.0014 | 0.0051 ± 0.006 | 0.14 | 0.50 |
| *Akkermansia muciniphila* | 0.43 ± 0.29 | 0.69 ± 0.35 | 0.30 ± 0.12 | 0.30^b^ | 0.47^b^ |
| *Prevotella* | 0.62 ± 0.41 | 0.85 ± 0.63 | 0.65 ± 0.40 | 0.46^b^ | 0.84^b^ |
| *Faecalibacterium prausitzii* | 3.20 ± 1.09 | 2.77 ± 1.07 | 2.53 ± 0.89 | 0.76 | 0.83^b^ |

^a^ qPCR, quantative polymerase chain reaction; DNA, deoxyribonucleic acid; PD, Parkinson’s disease. Control and baseline stool samples were collected during the baseline period. Stool samples were collected at week 5 of the intervention for the PD group only. Values represent mean ± standard error of the mean. Participants with PD at baseline were matched with controls and compared using paired t-test. Baseline vs. Intervention for the PD group was also compared using a paired t-test, unless otherwise indicated.

^b^ Compared using the non-parametric Wilcoxon Signed Rank test

**REFERENCES**

1. Papadaki A, Johnson L, Toumpakari Z, England C, Rai M, Toms S, et al. Validation of the English Version of the 14-Item Mediterranean Diet Adherence Screener of the PREDIMED Study, in People at High Cardiovascular Risk in the UK. *Nutrients* (2018) 10(2). Epub 2018/02/01. doi: 10.3390/nu10020138.

2. Schröder H, Fitó M, Estruch R, Martínez-González MA, Corella D, Salas-Salvadó J, et al. A short screener is valid for assessing Mediterranean diet adherence among older Spanish men and women. *J Nutr* (2011) 141(6):1140-5. Epub 2011/04/22. doi: 10.3945/jn.110.135566.

3. Postuma RB, Berg D, Stern M, Poewe W, Olanow CW, Oertel W, et al. MDS clinical diagnostic criteria for Parkinson's disease. *Movement disorders* (2015) 30(12):1591-601.

4. Lim H-S, Park S-K, Kwak I-S, Kim H-I, Sung J-H, Jang S-J, et al. HPLC-MS/MS analysis of 9 artificial sweeteners in imported foods. *Food Science and Biotechnology* (2013) 22(1):233-40. doi: 10.1007/s10068-013-0072-2.

5. Zygler A, Wasik A, Kot-Wasik A, Namieśnik J. Determination of nine high-intensity sweeteners in various foods by high-performance liquid chromatography with mass spectrometric detection. *Anal Bioanal Chem* (2011) 400(7):2159-72. Epub 2011/04/05. doi: 10.1007/s00216-011-4937-z.

6. Subar AF, Kirkpatrick SI, Mittl B, Zimmerman TP, Thompson FE, Bingley C, et al. The Automated Self-Administered 24-hour dietary recall (ASA24): a resource for researchers, clinicians, and educators from the National Cancer Institute. *J Acad Nutr Diet* (2012) 112(8):1134-7. Epub 2012/06/19. doi: 10.1016/j.jand.2012.04.016.
